# At Least 23 Genera Instead of One: The Case of *Iris* L. s.l. (Iridaceae)

**DOI:** 10.1371/journal.pone.0106459

**Published:** 2014-08-29

**Authors:** Evgeny V. Mavrodiev, Mario Martínez-Azorín, Peter Dranishnikov, Manuel B. Crespo

**Affiliations:** 1 Florida Museum of Natural History, University of Florida, Gainesville, Florida, United States of America; 2 Institute of Plant Science, Karl-Franzens University, Graz, Austria; 3 Buchholz High School, Gainesville, Florida, United States of America; 4 CIBIO (Institute of Biodiversity), University of Alicante, Alicante, Spain; The National Orchid Conservation Center of China; The Orchid Conservation & Research Center of Shenzhen, China

## Abstract

**Background:**

*Iris* L. s.l. is one of the most diverse and well-known genera in the Asparagales, with approximately 250–300 circumscribed species and significant economic impact. The taxonomy of the genus has suffered dramatic changes in the last century, particularly in the last decades after the application of molecular techniques. As a result several contrasting systematic arrangements are currently available to taxonomists. Many genera that were split from *Iris* s.str. in the past, on the basis of morphology (e.g., *Hermodactylus*, *Iridodictyum*, *Juno*, *Pardanthopsis*, and *Xiphion*, among others), are now *a priori* re-included in a very widely circumscribed *Iris* s.l. (incl. *Belamcanda*). This resulted in a more heterogeneous genus that is more difficult to define on morphological grounds. Testing congruence between taxonomic treatments and the results of recent molecular studies of *Iris* has never been performed, mostly due to the lack of proper taxonomic context.

**Results:**

We generated several conventional phylogenies for *Iris* & outgroups using extensive sampling of taxa (187) and characters (10 plastid loci). We demonstrate that the natural history of *Iris*, written either as conventional molecular phylogenies or, if viewing in the context of the comparative approach, as a nested most parsimonious hierarchy of patterns, appear to be fully congruent with the narrow taxonomical treatment of the genus, restricted to the rhizomatous “bearded” taxa. The resulting topologies place *Belamcanda*, *Pardanthopsis*, and *Gattenhofia* as sisters to *Iris* s.str. and genus *Siphonostylis* as sister to *Iris* s.l.

**Conclusion:**

The present study clearly justifies the splitting of *Iris* s.l. into at least 23 genera, 18 of which have already been accepted in the past by numerous authorities. These genera are characterized by unique combinations of partly overlapping morphological characters and biogeography. Moreover, nearly the same entities, which we here recognize at a generic rank, were for centuries frequently referred to by horticulturists as “working-name” groups.

## Introduction

With approximately 250–300 species in circumscribtion, *Iris* s.l. is one of the most diverse and well-known genera in the Asparagales. The genus also includes a few outstanding model systems in evolutionary biology, particularly those used for studying hybridization and speciation in plants (e.g., [1], [2]). Due to its popularity in the horticultural trade, *Iris* has significant economic impact. However the taxonomy of *Iris* s.l. remains complicated. Based on morphology, many genera were split from *Iris* s.str. and were widely accepted in the past (e.g. *Hermodactylus*, *Iridodictyum*, *Juno*, or *Xiphion*, among others. They are now *a priori* re-included in a widely circumscribed *Iris* s.l., which renders it more heterogeneous and difficult to define on morphological grounds.

The test for congruence of *Iris*’s taxonomy, with the results of recent molecular studies of *Iris*, seems to be critical, but it has never been performed in a proper way, mostly due to the lack of correct taxonomic context. Here, we present the phylogenies for the *Iris* s.l. & outgroups by using extensive sampling of taxa (187) and characters (10 plastid loci), establishing the largest molecular matrix yet assembled for the group.

We also paired conventional phylogenetic analyses with the three-taxon analysis (3TA) [3], [4], [5] of binary representations of DNA matrices of the *Iris* s.l. & outgroups.

We compare the obtained conventional molecular phylogenies of *Iris* and the most parsimonious hierarchy of patterns yielded by the three-taxon analyses, with the different taxonomical treatments of the genus, and propose a new taxonomic arrangement of *Iris* s.l.

## Results

**Figure 1:**
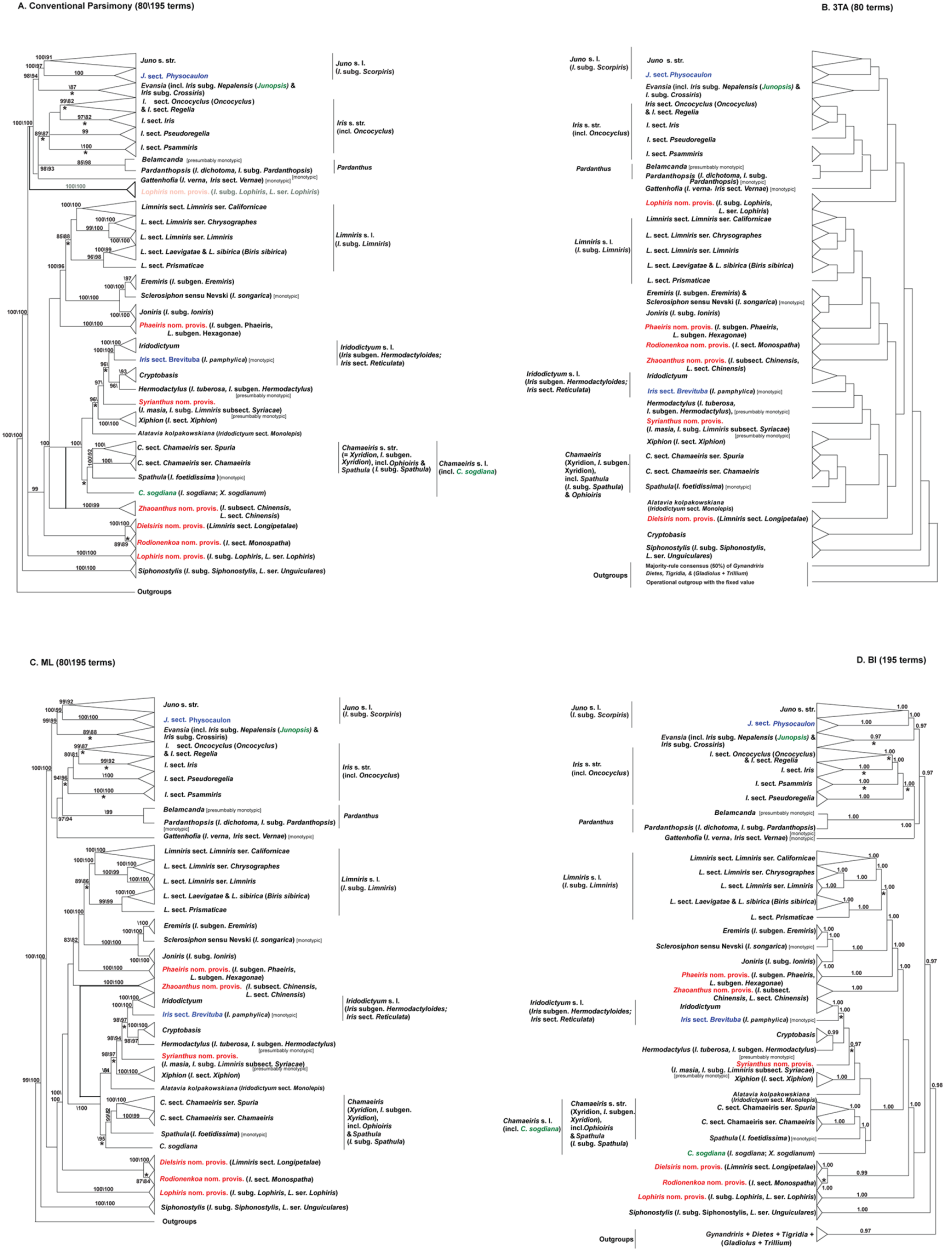
The summary of analyses. **A.** The summary of strict consensus of 24\160 (80\195 terms) most parsimonious topologies recovered from a MP analysis (PAUP*) of conventional *Iris* s.l. & outgroups plastid supermatrix. Bold branches show the positions of *Lophiris* nom. provis. (*Iris* subg. *Lophiris*) and *Zhaoanthus* nom. provis. (*I*. subsect. *Chinensis*) within the 80-term topology. See Figure S1 and Figure S2A for the details. **B.** The summary of the single most parsimonious topology recovered from a MP analysis (PAUP*) of WS representation of conventional *Iris* s.l. & outgroups plastid supermatrix (81 terms, 80 taxa (79 of *Iris* s.l. +1 outgroup) + operational outgroup). See Figure S3 for the details. **C.** Summary of the two most probable topologies (80\195 terms) recovered from a ML analysis (RAxML) of conventional *Iris* s.l. & outgroups plastid supermatrix. ML BS values for nodes receiving >80% supports are indicated above and below the branches. Bold branches show the position of *Zhaoanthus* nom. provis. (*I*. subsect. *Chinensis*) within 80-term topology. See Figure 2A and Figure S2B for the details. **D.** Consensus topology recovered from a Bayesian analysis (MrBayes) of conventional *Iris* s.l. & outgroups plastid supermatrix (195 terms). Numbers above and below branches indicate posterior probabilities >0.95. See Figure S4 for the details. Taxa, proposed to be accepted at the generic rank for the first time are indicated in red; taxa potentially recognizable at generic rank are indicated in blue, critical taxa are indicated in green. Selected synonyms of accepted or proposed genera are indicated in curved brackets. Asterisks indicate the branches with a minor conflict of support levels. The widely used name “*Limniris* (Tausch) Rchb.” must be conserved against “*Biris* Medik.” (*Iris sibirica* L.), as it was already conserved against “Pseudo-iris Medik.” [42].

**Figure 2:**
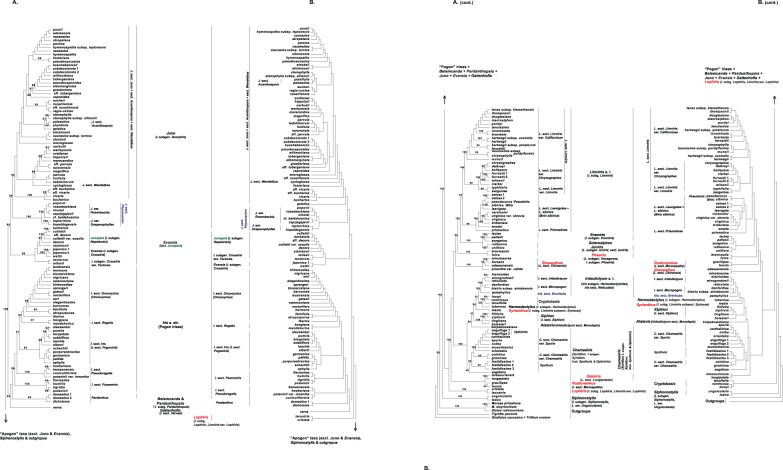
Conventional plastid phylogeny and nested most parsimonious hierarchy of patterns. **A.** Most probable topology (-ln likelihood = 45410.319704) recovered from a ML analysis (RAxML) of conventional *Iris* s.l. & outgroups plastid supermatrix (195 terms). ML BS values for nodes receiving >80% supports are indicated above and below the branches. **B.** Robinson-Foulds 3TA topology (RFS) of score 7 combined the results of all 3TA analyses (Figure S3 (single MP topology), Figure S5A (single MP topology), and Figures S5B–E (RF median consensus of the even numbers of MP topologies)). See Figure S3 and Figure S5 for the details. Taxa proposed to be accepted at the generic rank for the first time are indicated in red; taxa potentially recognizable at generic rank are indicated in blue, critical taxa are indicated in green. Selected synonyms of accepted or proposed genera are indicated in curved brackets.

Figure 1 provides the detailed summary of the results. The names of the clades are given in italics due to the strong congruence with various taxonomic entities. The phylogenetic analyses of either the complete or modified supermatrix and the three-taxon statements (3TSs) binary matrices yielded similar topologies with all of the traditional infrageneric taxa of *Iris* s.l., resolved as well or strongly supported monophyletic groups or lineages (Figures 1–2, Figures S1–S6).

Positions of monophyletic *Cryptobasis* Nevski (*I*. subsect. *Tenuifoliae* Diels), *I*. sect. *Psammiris* (Spach) J.J.Taylor (*I*. subgen. *Psammiris* Spach), *I*. sect. *Pseudoregelia* Dykes (*I*. subgen. *Pseudoevansia* Baker), *Lophiris* nom. provis. (*Iris* subg. *Lophiris* (Tausch) C.A. Wilson), *I*. subgen. *Crossiris* Spach (*I. watti* Baker ex Hook.f. + *I. japonica* Thunb.), *Juno* sect. *Acanthospora* Rodion., *J*. sect. *Wendelboa* Rodion., *Rodionenkoa* nom. provis. (*I.* sect. *Monospatha* Rodion.), *Spathula* (Tausch) Fourr. (*I. foetidissima* L., *I*. subg. *Spathula* (Tausch) Spach), and *Zhaoanthus* nom. provis. (*I*. subsect. *Chinensis* Diels, *Limniris* sect. *Chinensis* (Diels) Rodion.) depend on the chosen method of the analysis (Figures 1–2, Figures S1–S6).

Clade {*Pardanthus* (*Belamcanda* Adans. (*B. chinensis* (L.) Redouté, *I. domestica* (L.) Goldblatt & Mabb.) + *Pardanthopsis* (Hance) L.W. Lenz (*Pardanthopsis dichotoma* (Pall.) L.W. Lenz, *Iris dichotoma* Pall., *Pardanthus dichotomus* (Pall.) Ledeb.)} and *Gattenhofia* Medik. (*I*. subsect. Vernae Diels) are sister groups of the well or strongly supported *Iris* s.str. (Figures 1–2, Figure S1). Siphonostylis Wern. Schulze (*I.* subg. Siphonostylis (Wern. Schulze) C.A. Wilson (I. ser. Unguiculares *(*Diels) G.H.M. Lawr. = ***Limniris*** sect. *Unguiculares* (Diels) Rodion.)), was confirmed as the sister group to the rest of *Iris* s.l. (Figure 1, Figures S1–S2).

*Evansia* Salisb. (incl. *Junopsis* Wern.Schulze (*I.* subg. *Nepalensis* (Dykes) G.H.M. Lawr.)) is sister to *Juno* Tratt. (*I*. subgen. *Scorpiris* Spach), and {*Juno* + *Evansia*} + {*Iris* s.str. + *Gattenhofia* + *Belamcanda* + *Pardanthopsis*} are strongly supported sister clades.

The monotypic genus *Sclerosiphon* Nevski (*Iris songarica* Schrenk) is a strongly supported sister to *Eremiris* (Spach) Rodion. (*I.* subgen. *Eremiris* Spach) and both latter groups form a a strongly supported sister clade to *Joniris* (Spach) Klatt (*I.* subg. *Ioniris* Spach). Clade {*Sclerosiphon + Eremiris + Joniris*} is a strongly supported sister of *Limniris* (Tausch) Rchb. s.l.

*Iridodictyum* Rodion (*Iris* subgen. *Hermodactyloides* Spach; *I*. sect. *Reticulata* Dykes) (incl. *Iris* sect. *Brevituba* B. Mathew (*I. pamphylica* Hedge)), (*Cryptobasis* Nevski (conventional phylogenies only) + *Hermodactylus* Mill. (*I*. subgen. *Hermodactylus* (Tourn.) Sweet (*I. tuberosa* L.)), *Syrianthus* nom. provis. (*I. masia* Dykes, *I*. subg. *Limniris* (Tausch) Spach subsect. *Syriacae* Diels), *Xiphion* Mill. (*I*. sect. *Xiphion* (Mill.) Tausch), *Alatavia kolpakowskiana* (Regel) Rodion. (*Iridodictyum* sect. *Monolepis* Rodion., *Iris kolpakowskiana* Regel), and *Chamaeiris* Medik. (*Xyridion* (Tausch) Fourr., *I*. subgen. *Xyridion* Spach (incl. *Spathula*)) formed a grade with the partly conflict levels of support (Figure 1–2, Figures S1–S6).

*Juno* sect. *Physocaulon* Rodion. is a strongly supported sister clade to the rest of the *Juno*, *I*. sect. *Brevituba* (*I. pamphylica*) is strongly supported sister to *Iridodictyum*, and *Chamaeiris sogdiana* (Bunge) M.B.Crespo (*Iris sogdiana* Bunge, *Xyridion sogdianum* (Bunge) Nevski) is a sister to the rest of the *Chamaeiris* (Figure 1–2, Figures S1–S6).

Several species sampled in more than one infraspecific taxa, appeared to be non-monophyletic (*I. caucasica* Hoffm., *I. hartwegii* Baker, *I. potaninii* Maxim. and others) (Figure 2).

## Analyses

We sampled 173 broadly defined species of *Iris* s.l. and five out-group taxa: *Dietes* Salisb. (*D. robinsoniana* Klatt), *Gladiolus* L. (*G. caucasicus* Herb.), *Gynandriris* Parl. (*G. pritzeliana* (Diels) Goldblatt and *G. sisyrinchium* Parl.), *Tigridia* Juss. (*T. pavonia* (L.f.) DC.), and *Trillium* L. (*T. ovatum* Pursh) (Appendix S1). For seven species (*Iris anguifuga* Y.T. Zhao & X.J. Xue, *I. foetidissima* L., *I. domestica* (L.) Goldblatt & Mabb., *I. japonica* Thunb., *I. forrestii* Dykes, *I. setosa* Pall. ex Link, and *I. subdecolorata* Vved.) (Appendix S1) two or three accessions were included in the analyses. For 10 species (*I. caucasica*, *I. collettii* Hook.f., *I. hartwegii* s.l. (incl. *I. pinetorum* Eastw.), *I. histrio* Rchb.f., *I. hymenospatha* B. Mathew & Wendelbo, *I. potaninii*, *I. proantha* Diels, *I. stenophylla* Hausskn. ex Baker, *I. tenuissima* Dykes, and *I. virginica* L. s.l. (incl. *I. shrevei* Small) infraspecific taxa (either subspecies or varieties) were included in the analyses (Appendix S1). With the exclusion of *Alatavia*, all *Iris*-segregated genera, as well as a vast majority of the infrageneric groups of the broadly defined *Iris*, were sampled in two or more taxa (if not monotypic) (Appendix S1). The total number of taxa sampled for *Iris* s.l. & outgroups was 187 (182 for *Iris* s.l.).

The sequence data (10 plastid loci: 5′ *trnK*, *matK*, 3′ *trnK*, *trnL* intron, *trnL-F* IGS, *ndhF*, *rpl14*-r*ps8* IGS, *rps8* gene, *rps8-rpL36* IGS, and *trnE-trnT* spacer) was taken from the GenBank/EMBL databases (Appendix S1). Sequences were generated mostly by [6], [7], [8], [9], [10], [11], [12], [13], [14], [15], [16], [17], [18], [19] (Appendix S1), and in the majority during the long-term comprehensive studies of Wilson [15], [16], [17] and Ikinci et al. [18]. *Iris domestica, I. anguifuga*, *I. falcifolia* Bunge, *I. foetidissima*, *I. loczyi* Kanitz, *I. pallasii* Fisch. ex Trevir., *I. tenuifolia* Pall., and *I. ventricosa* Pall. were sampled, additionally sampled or re-sampled from [7], [12], [13], [18], [19] (Appendix S1) (see also [18] for the brief discussion on *I. falcifolia*). Following [20] and [21], the sequence data for *trnE*-*trnT* and *rpl14-rps8* spacers of *Trillium ovatum* [14] were combined with the sequence data of the most distant *Iris*’s s.l. outgroup (*Gladiolus*).

All sequences were aligned using MAFFT [22], [23], and then were concatenated and analyzed as a single contiguous dataset (supermatrix). The number of terms in the final supermatrix was 195. We followed MAFFT’s FFT-NS-i, E-INS-i, L-INS-i, and G-INS-i alignment strategies [22], [23], with the default settings for gap opening penalty and offset value. Including gaps, the total G-INS-i alignment used for the final analyses consisted of 8464 bp.

Five analytical approaches were used:

1. Bayesian analyses (BI) of the 195 term supermatrix were conducted with the MrBayes (v. 3.1.2) [24]. Two runs with four chains each (three heated and one cold) were run for 40 million generations; the chains were sampled every 1000 generations with default parameters.
2. We analyzed the 80 and 195 term supermatrces by the maximum likelihood (ML) approach, as implemented in RAxML v. 7.4.2 [25], [26] with 2000 rapid bootstrap (BS) replicates, integrated with 200 searches for the optimal tree.
3. The 195 term supermatrix was also analyzed by PhyML v. 3.1 [27], as implemented in SeaView v. 4.5.1 [28], with estimated proportion of invariable sites and empirical nucleotide equilibrium frequencies. We took a BioNJ tree as a starting tree, and defined the strategy of the tree topology search as “best of NNIs and SPRs” [27]. Instead of the ML BS, branch supports were calculated with the approximate likelihood-ratio test (aLRT) [29].

In the cases of parametric approaches, the GTR + G model was assumed to be the best choice.

1. Conventional maximum parsimony (MP) analysis of both the 80 and 195 term supermatrices, was performed with PAUP* v. 4.0b10 and 4.0a134 [30], using heuristic searches with 1000 random addition replicates, with no more than 100 trees saved per replicate, and tree-bisection-reconnection (TBR) branch swapping with the MulTrees option in effect. MP Jackknife (JK) values of clade support are estimated using 2500 replicates and 10 random addition sequences (saving no more than 1000 trees per replicate), with the TBR branch swapping/MulTrees option in effect with the deletion of 37.0% of the characters in each replicate.
2. The three-taxon analysis (3TA) of the DNA matrices was established after their three-taxon Williams-Siebert (WS) representation [31], [32] using TAXODIUM v. 1.2 [32]. The value of the operational outgroup was fixed as a consensus sequence of the matrix [*Dietes* + *Gynandriris* + *Tigridia* + (*Gladiolus* + *Trillium*)], or, in some cases, as a consensus of matrices [*Iris japonica* 1, 2 + *I. watti*] (Figure S5B) or [*Belamcanda* (*I. domestica*) + *Pardanthopsis* (*I. dichotoma*) + *Gattenhofia* (*I. verna*)] (Figure S5C). The majority rule consensus (50%) was used for the calculation of the consensus sequences (only modal values, shown with the minimum frequency among applicable states, required to include the state in consensus equal to 0.5).

The WS or binary representation of the DNA matrix is, in fact, the 3TS matrix [32]. Therefore below, we use the term “WS representation” as a synonym of the term “3TS matrix”.

Due to the computational limitations for the MP search, before WS representation, the conventional matrix of *Iris* s.l. & outgroups was reduced down to 80 taxa (79 species + single outgroup), but retained the sampling of all major taxonomic entities (Figure 1B, Figure S3). Based on the relationships obtained after the MP analysis of this 3TS matrix, five additional “local” [33] 3TAs were performed, each *within* [33] one of the fully sampled major clades of the obtained 3TA topology (Figure S5).

With the exclusion of a single most parsimonious topology, which was recovered after the MP analysis of the 3TS matrix of *Evansia* (Figure 5SB), the even number of the most parsimonious trees was obtained after each local MP search (Figure 5S). In all cases, the topology of strict consensus was not minimal. Therefore, additionally to the strict consensus [4], [5], we calculated the median consensus tree (reviewed in [5], [34]), based on Robinson-Foulds (RF) distance [34]. Calculations were performed by using RFS v. 2.0 [34] (Figures S5A, C–E).

Eventually all six minimal 3TA topologies (two single, most parsimonious trees (Figure S3, S5A) and four RF median consensus, each represented one of the minimal trees (Figures S5A, C–E)) were combined to the single median RF Supertree [34] (Figure 2B), and additionally to the almost identical, single, median Supertriplets-based supertree [35] (not shown).

In cases of all 3TAs, we used the uniform weighing (reviewed in [4], [5]) of the statements. The results of the 3TAs were accepted as preliminary, but sufficient to the comparison with conventional phylogenies (Figures 1–2).

Trees and matrices were handled using Se-Al v. 2.0a11 [36], Mesquite v. 2. 75 [37], SeaView v. 4.5.1 [28], and FigTree v. 1.4 [38]. Resources of bioinformatics portal CIPRES (https://www.phylo.org/) and RCC of University of Florida (http://researchcomputing.ufl.edu/), were used for the several MP and BI analyses.

## Discussion

Linnaeus [39] accepted a broadly defined genus *Iris*, contrary to previous authors such as Bauhin and Cherler [40], Dodoens [41], Tournefort [42] among others. However, almost at the same time, Miller [43], [44], and later Adanson [45], Fourreau [46], Medikus [47], Parlatore [48], Reichenbach [49], and Trattinnick [50] among others, challenged Linnaeus’s treatment, by accepting segregation of additional genera [39], [40], [41]. Linnaean’s “*Iris* s.l.”, however, appeared to be normative for most experts until today, despite the fact that a broad definition of *Iris*, as currently circumscribed, makes that group too heterogeneous, and therefore difficult to define [51], [52], [53].

Molecular contributions of Tille et al. [54] and Wilson [15], [16], [17] have demonstrated that *Belamcanda chinensis* is deeply nested within the *Iris* s.l. clade. Thus, in case of recognition of *Belamcanda* as an independent genus, *Iris* s.l. appears to be clearly non-monophyletic [15], [16], [17], [54]. Therefore, in the light of the recent molecular data [15], [16], [17], [18], to make *Iris* s.l. monophyletic, all bulbous genera, namely the frequently recognized *Alatavia*, *Iridodictyum*, *Juno*, and *Xiphion*, plus the rhizomatous or tuberous *Chamaeiris*, *Hermodactylus* or *Junopsis*, must be circumscribed within *Iris.* The presumably monotypic, polypoid genera *Belamcanda* and *Pardanthopsis* must also be circumscribed within *Iris*, but this matter seems to be very problematic [55]. For example, Lenz [56], [57], [58] and Schulze [59] listed about a dozen morphological, anatomical and biological features, which clearly separate *Pardanthopsis* and *Belamcanda* from *Iris* s.l., as well as from each other. These authors also showed that both genera do not form hybrids with other species of *Iris* s.l., but can mutually interbreed to produce × *Pardancanda norrisii* L.W. Lenz (*I*. × *norrisii* (L.W. Lenz) C. Whitehouse) [56], [58]. Attention must also be paid to the fact that the basionym of *Belamcanda chinensis* is *Ixia chinensis* L., a name applied to plants with an actinomorphic open flower, which is clearly different from the typical 3-merianthic, closed *Iris*-flower structure.

Strong morphological evidence has been used to argue in favor for separating several genera from *Iris* s.str., such as *Alatavia* [60], *Chamaeiris* (*Xyridion*) [51], [61], *Cryptobasis* [62], [63], [64], [65], [66], *Eremiris* [67], *Iridodictyum* [68], *Juno* [68], [69], *Limniris* [70], *Sclerosiphon* [66], *Siphonostylis* [71], [72], [73], and *Xiphion* [68], [74]. They all constitute independent lineages, which are easy to define on morphological grounds. Among these, natural hybridization is almost unknown to occur, aside from one to a few potentially credible cases (e. g., *Iris* × *neumayeri* Janch. ex Holub (*I*. *graminea* L. × *I. sibirica* L.)).

Our data are essentially congruent with the results of Shneer’s [75] (see also [76] and [77]), who showed that serologically *Hermodactylus*, *Gynandriris*, *Iris*, *Xiphion*, *Juno*, *Pardanthopsis*, *Iridodictyum*, and *Belamcanda* are nested into two groups: (a.) *Hermodactylus* + *Gynandriris* on one side, and (b.) the rest of the genera on the other. Within group (b.), the “beardless” irises (*Limniris* s.l.) are sharply different from the “bearded” irises (*Iris* s.str.), which are serologically closer to *Juno*, *Pardanthopsis*, and *Belamcanda*. In contrast, *Chamaeiris* (*Xyridion*), *Iridodictyum*, and *Xiphion*, appeared to be more closely related to the “beardless” irises (*Limniris* s.l.), not to *Iris* s.str. [75].

Later Shneer [78] and Rodionenko [66], [71] also argued that the *Iris* sect. *Unguiculares* is a separate genus, *Siphonostylis* [72], [73]. According to Shneer [78] and Rodionenko [66], [71], *Siphonostylis* displayed features closer to those present in presumably primitive irises. Accordingly, in our analyses, this later genus is the sister group to the rest of the broadly defined *Iris* s.l. clade (Figure 1, Figures S1–S3, see also [16], [17], [55] for similar results). Also, Shneer [78] confirmed that serologically *Iris* s.str. and the “beardless” taxa (*I.* subg. *Limniris* and *I.* subg. *Xyridion*, both accepted here at the generic rank as *Limniris* and *Chamaeiris* respectively) are very dissimilar, and she also showed [78] that the members of the relatively homogeneous “*Iris* s.str.”, appear to be similar to irises of *I.* subg. *Crossiris*, the genus *Evansia* (incl. *Junopsis* [79]) of our topologies (Figure 1, Figures S1–S2A).

*Belamcanda* and *Pardanthopsis* are sister taxa to each other, and therefore may be treated as either one or two genera. Due to the agreement that *Belamcanda* and *Pardanthopsis* must both be accepted at the generic rank [56], [58], [80], we tend to agree with the morphologically diverse genus × *Pardancanda* W. Lenz which results from the artificial crossing of the closely related *Belamcanda* and *Pardanthopsis* [56], [58].

Our findings do not mean, however, that all *Iris* - segregated genera, are congruent to our topologies. Due to the recent sampling of sequence data, we did not find enough evidence to accept genera such as *Biris* Medik, *Junopsis*, *Neubeckia* Alef., *Oncocyclus* Siemssen, *Regelia* Hort. ex H. Wendl., *Spathula*, and *Ophioiris* (Y.T. Zhao) Rodion. [81], but at least the case of *Junopsis* [82] clearly requires further investigation.

The re-treated results of recent comprehensive molecular studies of *Iris* s.l. [15], [16], [17], [18], if placed in a proper taxonomic context, provide unique opportunity to show that a rainbow cannot consist of a single color, and a broadly defined *Iris* is better treated as a tribe rather than a single genus. The natural history of *Iris*, written either as conventional molecular phylogenies (Figure 1–2, Figures S1, 2S, S4, S6) or, if viewing in the context of the comparative approach, as a nested most parsimonious hierarchy of patterns [4], [83] implying the “fourth parallelism” [84] (Figure 1–2, Figures S3, S5), appear to be fully congruent with the narrow taxonomical treatment of the genus, restricted to the rhizomatous “bearded” taxa. This leads to a new taxonomic arrangement of the whole aggregate, with at least 23 previously recognized infrageneric groups needing to be accepted at the generic rank (Figures 1–2). At least 18 of these groups have already been treated as independent genera by different authorities in the past (Figure 1–2, Figures S1–S6, Table S1), and many of them are still in current use. Our multi-generic proposal for *Iris* s.l. (Crespo et al., in prep.) is mostly concurrent with the distinction of groups, which have traditionally been used (and are still currently used) as “working-names” by horticulturists [85] within the last two centuries. It renders a more simple and practical nomenclatural system, than an alternative complex treatment of an expanded *Iris* with numerous infrageneric taxa. Unique combinations of partly overlapped morphological characters can be successfully used as a diagnostic for taxonomic recognition of such smaller and then more homogeneous and intuitively clear genera (Figures 1–2, Table S1).

In his early classification of the Old World *Iridoideae*, Goldblatt [86] suggested that the genera *Dietes*, *Iris* s.l., *Hermodactylus*, and *Belamcanda* should be grouped into one subtribe *Iridinae* Pax. This is generally supported in our present study.

Our multi-generic solution of *Iris* s.l. parallels the new recently proposed classifications of Hyacinthaceae subfam. Ornithogaloideae (Asparagaceae subfam. Scilloideae tribe Ornithogaleae) [87], *Typha* L. (Typhaceae) [88], [89], *Chenopodium* L. (Chenopodiaceae-Amaranthaceae) [90], *Aloe* L. (Xanthorrhoeaceae subfam. Asphodeloideae) [91], *Nothofagus* Blume [92] (Nothofagaceae), or or *Centaurium* Hill [93] (Gentianaceae), which simplify the taxonomy of the whole aggregate, and makes all segregate genera more homogeneous and easy to work with.

A broadly defined *Iris* seems to be semantically equivalent to tribe *Hordeeae* Kunth ex Spenn. & Martynov (former *Triticeae* Dumort.) with its sometimes cryptic, sometimes clear morphological diversity, wide range of chromosome numbers, and polyploid complexes (Table S1). The inclusion of *Belamcanda*, as well as of numerous other *Iris*-segregated genera in the circumscription of *Iris* s.l, may therefore be similar to the recognition of the tribe Hordeeae, at the rank of single genus - for example, the genus *Hordeum* L. s.l. It is hard to imagine, however, that such a decision will not be challenged, even if the pro-arguments will be nominated as practical expediency, problems with the nomenclature, and as other third-party considerations.

## Supporting Information

Figure S1 **Conventional plastid phylogeny (MP).** Strict consensus of 160 most parsimonious topologies (length = 5268, CI = 0.6137, RI = 0.8365) recovered from a MP analysis (PAUP*) of conventional of *Iris* s.l. & outgroups plastid supermatrix (195 terms, 8464 characters in total, 1559 are parsimony-informative). All characters were treated as “unordered” (Fitch parsimony). MP JK values for nodes with greater than 80% support are indicated above or below the branches.
(EPS)

Figure S2 **Conventional plastid phylogenies (reduced supermatrix). A.** Strict consensus of 24 most parsimonious topologies (length = 3512, CI = 0.6723, RI = 0.7695) recovered from a MP analysis (PAUP*) of conventional of *Iris* s.l. & outgroups plastid supermatrix (80 terms, 8237 characters in total, 1045 characters are parsimony-informative). All characters were treated as “unordered” (Fitch parsimony). MP JK values for nodes with greater than 80% support are indicated above or below the branches. **B**. Most probable topology (−ln likelihood = 33243.302540) recovered from a ML analysis (RAxML) of conventional *Iris* s.l. & outgroups plastid supermatrix (80 terms, 8237 characters). ML BS values for nodes receiving >80% supports are indicated above and below the branches. Taxa proposed to be accepted at the generic rank for the first time are indicated in red; taxa potentially recognizable at generic rank are indicated in blue; critical taxa are indicated in green.
(EPS)

Figure S3 **Nested most parsimonious hierarchy of patterns (reduced supermatrix).** Single most parsimonious topology (length = 2558783; RI = 0.7858) recovered from the MP analysis (PAUP*) of WS representation of conventional *Iris* s.l. & outgroups plastid supermatrix (81 terms, 80 taxa (79 taxa of *Iris* s.l. + outgroup ( = majority-rule consensus (50%) of the matrix [*Gynandriris* + *Dietes* + *Tigridia* + (*Gladiolus* + *Trillium*)]) + operational outgroup). All 2 023 963 binary characters (3TSs) are parsimony-informative, all weighted uniformly and all treated as “ordered” (Wagner parsimony). The value of operational outgroup fixed as a value of majority-rule consensus (50%) of the matrix [*Gynandriris* + *Dietes* + *Tigridia* + (*Gladiolus* + *Trillium*)].
(EPS)

Figure S4 **Conventional plastid phylogeny (BI).** Consensus topology recovered from a Bayesian analysis (MrBayes) of conventional *Iris* s.l. & outgroups plastid supermatrix (195 terms, 8464 characters in total). The first 3000 trees were discarded as burn-in, and posterior probabilities were calculated from the majority-rule consensus of the remaining trees sampled in both runs. At the end of the runs, the standard deviation of split frequencies between the two runs had fallen to 0.0070. Numbers above and below the branches indicate posterior probabilities >0.95. Taxa, proposed to be accepted at the generic rank for the first time are indicated in red; taxa potentially recognizable at generic rank are indicated in blue; critical taxa are indicated in green.
(EPS)

Figure S5 **Nested most parsimonious hierarchies of patterns. A.** 1. Strict consensus of the four most parsimonious topologies (length = 700315; RI = 0.8943; the length of the strict consensus equals to 700319) recovered from the MP analysis (PAUP*) of the WS representation of conventional plastid supermatrix *Juno* (57 terms). All 633358 binary characters (3TSs) are parsimony-informative, all are weighted uniformly and all are treated as “ordered” (Wagner parsimony). The value of the operational outgroup is fixed as a value of the majority-rule consensus (50%) of the matrix [*Gynandriris* + *Dietes* + *Tigridia +* (*Gladiolus* + *Trillium*)]; 2. RF median consensus (RFS) of the same most parsimonious topologies (RF distance score = 46; length = 700315; RI = 0.8943). **B**. Single most parsimonious topology (length = 1279; RI = 0.9412) recovered from the MP analysis (PAUP*) of WS representation of the conventional, plastid supermatrix of *Evansia* (10 terms). All 1208 binary characters (3TSs) are parsimony-informative, all are weighted uniformly and all are treated as “ordered” (Wagner parsimony). Based on the patterns of the relationships recovered from a MP analysis of the 81 terms 3TA matrix (Figure S3), the value of the operational outgroup is fixed as a value of the majority-rule consensus of the matrix [*Iris japonica* 1, 2 + *I. wattii*]. **C**. 1. Strict consensus of the 90 most parsimonious topologies (length = 72792; RI = 0.9363; the length of the strict consensus equal to 72884) recovered from the MP analysis of WS representation of conventional plastid supermatrix of the core *Iris* (37 terms). All 68435 characters (3TSs) are parsimony-informative, all are weighted uniformly and all are treated as “ordered” (Wagner parsimony). Based on the patterns of the relationships recovered from a MP analysis of the 81 term 3TA matrix (Figure S3), the value of the operational outgroup is fixed as a value of the majority-rule consensus of the matrix [*Belamcanda* (*I. domestica*) + *Pardanthopsis* (*I. dichotoma*) + *Gattenhofia* (*I. verna*)]; 2. RF median consensus (RFS) of the same most parsimonious topologies (RF distance score = 824; length = 72792; RI = 0.9363). **D**. 1. Strict consensus of the two most parsimonious topologies (length = 364930; RI = 0.8704; the length of the strict consensus equal to 364936) recovered from the MP analysis (PAUP*) of WS representation of conventional plastid supermatrix of *Limniris* s.l. + *Phaeiris* nom. provis. (*L*. subgen. *Hexagonae*, *I*. subgen. *Phaeiris*) + *Eremiris* (*I*. subgen. *Eremiris*) + *Joniris* (*L*. subgen. *Ioniris*; sect. *Ioniris*) + *Sclerosiphon* (*I. songarica*) **(**41 terms). All 323073 characters (3TSs) are parsimony-informative, all are weighted uniformly and all are treated as “ordered” (Wagner parsimony). The value of the operational outgroup is fixed as a value of the majority-rule consensus (50%) of the matrix [*Gynandriris* + *Dietes* + *Tigridia* + (*Gladiolus* + *Trillium*)]; 2. RF median consensus (RFS) of the same most parsimonious topologies (RF distance score = 7; length = 364930; RI = 0.8704). **E**. 1. Strict consensus of the 18 most parsimonious topologies (length = 87337; RI = 0.8526; the length of the strict consensus equals to 87347) recovered from the MP analysis (PAUP*) of WS representation of the conventional plastid supermatrix of *Iridodictyum* s.l. (*Iris* subgen. *Hermodactyloides*; *Iris* sect. *Reticulata*) + *Hermodactylus* (*I*. subgen. *Hermodactylus*) + *Syrianthus* (*I*. subg. *Limniris* subsect. *Syriacae*) + *Xiphion* (*I*. subg. *Xiphion*) + *Alatavia* (*Iridodictyum* sect. *Monolepis*) + *Chamaeiris* (*Xyridion*; *I*. sect. *Xyridion*) (28 terms). All 76118 characters (3TSs) are parsimony-informative, all are weighted uniformly and all are treated as “ordered” (Wagner parsimony). The value of the operational outgroup is fixed as a value of the majority-rule consensus (50%) of the matrix [*Gynandriris* + *Dietes* + *Tigridia* + (*Gladiolus* + *Trillium*)]; 2. RF median consensus (RFS) of the same most parsimonious topologies (RF distance score = 0; length = 87337; RI = 0.8526).
(EPS)

Figure S6 **Conventional plastid phylogeny (ML).** Most probable topology (-ln likelihood = 45051.530110) recovered from a ML analysis (PhyML) of the conventional *Iris* s.l. & outgroups plastid supermatrix (195 terms, 8464 characters in total). The aLRT support values of 0.9 or higher are indicated above and below the branches. Taxa proposed to be accepted at the generic rank for the first time are indicated in red; taxa potentially recognizable at generic rank are indicated in blue; critical taxa are indicated in green.
(EPS)

Table S1 **Summary of morphological characters and chromosomal counts for the accepted genera.**
(EPS)

Appendix S1 **List of taxa with GenBank-EMBL accession numbers used in the analyses.**
(PDF)
