# Supplementary material for: At Least 23 Genera Instead of One: The Case of Iris L. s.l. (Iridaceae)
Source: PLoS One. 2014 Aug 29;9(8):e106459. doi: 10.1371/journal.pone.0106459 (PMC4149580; doi:10.1371/journal.pone.0106459)
Supplement: Appendix S1 — List of taxa with GenBank-EMBL accession numbers used in the analyses. (PDF) [file pone.0106459.s008.pdf]

Appendix 1. List of taxa with GenBank-EMBL accession numbers used in the analyses*

[Loci: 5' *trnK*; *matK*; 3' *trnK*; *trnL* intron; *trnL-F* IGS; *ndhF*; *rpl14-rps8* IGS, *rps8* gene, & *rps8-rpL36* IGS; *trnE-trnT* spacer]

Iris aitchisonii (Baker) Boiss. FR870402 FR870402 FR870613 FR870613 FR870679 FR870547; *I. alberti* Regel HM574555 HM574680 HM574396; *I. albomarginata* R.C.Foster FR870413 FR870413 FR870624 FR870624 FR870690 FR870558; *I. alexeenkoi* Grossh. HM574556 HM574397; *I. anguifuga* Y.T.Zhao & X.J.Xue 1 JF954116; *I. anguifuga* Y.T.Zhao & X.J.Xue 2 JF954117; *I. aphylla* L. HM574546 AY596661 AY596661 HM574398; *I. atropatana* Grossh. FR870431 FR870431 FR870642 FR870642 FR870708 FR870576; *I. atropurpurea* Baker FR870386 FR870386 FR870597 FR870597 FR870663 FR870531; *I. aucheri* (Baker) Sealy HM574525 FR870384 FR870384 FR870661 FR870529; *I.* cf. *baldshuanica* O.Fedtsch. FR870400 FR870400 FR870611 FR870611 FR870677 FR870545; *I. barbatula* Noltie & K.Y.Guan HM574547 HM574647 HM574400; *I. barnumiae* Foster & Baker HM574547 AY596660 HM574612 HM574401; *I. bicapitata* Colas. HM574508 HM574402; *I. bismarckiana* Regel HM574403; *I. boissieri* Henriq. HM574523 HM574650 HM574404; *I. bostrensis* Mouterde HM574405; *I. bracteata* S.Watson FJ156284 FJ197221 EU939455 EU939455; *I. brevicaulis* Raf. FJ156285 AY596651 AY596651 EU939456 EU939456 HM574406; *I. bucharica* Foster FR870389 FR870389 FR870600 FR870600 FR870666 FR870534; *I.* aff. *bucharica* Foster FR870439 FR870439 FR870650 FR870650 FR870716 FR870584; *I. bulleyana* Dykes FJ156286 FJ197269 FJ197222 EU939457 EU939457; *I. capnoides* (Vved.) T.Hall & Seisums FR870418 FR870418 FR870629 FR870629 FR870695 FR870563; *I. carthaliniae* Fomin FJ156287 AY596637 AY596637 HM574407; *I. caucasica* Hoffm. HM574526 FR870377 FR870377 FR870588 FR870588 FR870654 FR870522; *I. caucasica* Hoffm. subsp. *turcica* B.Mathew HM574550 FR870429 FR870429 FR870640 FR870640 HM574408 FR870706 FR870574; *I. chrysographes* Dykes FJ156288 FJ197270 FJ197223 EU939459 EU939459; *I. chrysophylla* Howell FJ156289 FJ197271 FJ197224 EU939460 EU939460 HM574410; *I. clarkei* Baker ex Hook.f. FJ156290 FJ197272 FJ197225 EU939461 EU939461; *I. colchica* Kem.-Nath. FJ156291 AY596632 AY596632 EU939462 EU939462; *I. collettii* Hook.f. HM574521 AY596664 HM574587 HM574412; *I. collettii* Hook.f. var. acaulis Noltie FR870426 FR870426 FR870703 FR870571; *I. cristata* Aiton FJ156292 AY596639 AY596639 EU939463 EU939463 HM574413; *I. cuniculiformis* Noltie & K.Y.Guan AY596656 AY596656; *I. cycloglossa* Wendelbo HM574527 FR870390 FR870390 FR870601 FR870601 HM574414 FR870667 FR870535; *I. danfordiae* Boiss. FJ156293 AY596630 AY596630 EU939464 EU939464 HM574415; *I. decora* Wall. FJ156294 FR870434 FR870434 FR870645 FR870645 HM574416 FR870711 FR870579; *I.* aff. *decora* Wall. FR870425 FR870425 FR870636 FR870636 FR870702 FR870570; *I. delavayi* Micheli FJ156295 FJ197274 FJ197227 EU939466 EU939466 HM574417; *I. dichotoma* Pall. AJ579962 AJ579962 HM574418; *I. domestica* (L.) Goldblatt & Mabb. 1 AY596652 HM574603 HM574419; *I. domestica* (L.) Goldblatt & Mabb. 2 AJ579961 AJ579961; *I. douglasiana* Herb. FJ156296 KC118917 FJ197228 EU939467 EU939467; *I. edomensis* Sealy FR870416 FR870416 FR870627 FR870627 FR870693 FR870561; *I. elegantissima* Sosn. HM574553 AY596658 HM574617 HM574420; *I. ensata* Thunb. FJ156297 FJ197276 FJ197229 EU939468 EU939468 HM574421; *I. falcifolia* Bunge FR870421 FR870421 FR870632 FR870632 FR870698 FR870566; *I. fernaldii* R.C.Foster FJ156298 FJ197277 FJ197230 EU939469 EU939469; *I. filifolia* Bunge HM574522 HM574649 HM574422; *I. flavissima* Besser HM574513 HM574639 HM574424; *I. foetidissima* L. 1 FJ156299 FJ197278 FJ197231 EU939470 EU939470 HM574425; *I. foetidissima* L. 2 HE967421; *I. foetidissima* L. 3 JN895312; *I. forrestii* Dykes 1 FJ156300 AY596645 AY596645 EU939471 EU939471 HM574426; *I. forrestii* Dykes 2 JF954140; *I. fosteriana* Aitch. & Baker AY596671 FR870392 FR870669 FR870537; *I. fulva* Ker Gawl. FJ156301 AY596650 AY596650 EU939472 EU939472 HM574427; *I. galatica* Siehe FJ156302 FR870381 FR870381 EU939473 EU939473 FR870658 FR870526; *I. gatesii* Foster HM574559 HM574428; *I. germanica* L. HM574509 HM574636 HM574429; *I. hermona* Dinsm. HM574557 HM574682 HM574621 HM574434; *I. gracilipes* A.Gray HM574566 HM574430; *I. graeberiana* Sealy FR870432 FR870432 FR870643 FR870643 FR870709 FR870577; *I. graminea* L. HM574544 HM574670 HM574431; *I. hartwegii* Baker subsp. *australis* Parish FJ156303 FJ197280 FJ197233 EU939474 EU939474; *I. hartwegii* Baker FJ156304 FJ197281 FJ197234 EU939475 EU939475; *I. hartwegii* Baker subsp. *pinetorum* (Eastw.) L.W.Lenz FJ156305 FJ197282 FJ197235 EU939476 EU939476; *I. hippolyti* (Vved.) Kamelin FR870419 FR870419 FR870630 FR870630 FR870696 FR870564; *I. histrio* Rchb.f. subsp. *aintabensis* (G.P.Baker) B.Mathew HM574659 HM574435; *I. histrioides* Fost. ex Hayek AY596631 AY596631; *I. hoogiana* Dykes HM574517 HM574644 HM574436; *I. hookeriana* Foster HM574562 HM574687 HM574437; *I. humilis* Georgi HM574513 AY596655 HM574579 HM574438; *I. hymenospatha* B.Mathew & Wendelbo FR870436 FR870436 FR870647 FR870647 FR870713 FR870581; *I. hymenospatha* subsp. *leptoneura* B.Mathew & FR870385 FR870385 FR870596 FR870596

FR870662 FR870530; *I. iberica* Hoffm. AY596657 AY596657; *I. innominata* L.F.Hend. FJ156307 FJ197284 FJ197237 EU939478 EU939478 HM574439; *I. japonica* Thunb. 1 HM574563 FR870428 FR870428 FR870639 FR870639 HM574440 FR870705 FR870573; *I. japonica* Thunb. 2 AB088786; *I. kemaonensis* D.Don HM574519 HM574646 HM574441; *I. kirkwoodiae* Chaudhary HM574552 HM574442; *I. kolpakowskiana* Regel HM574533 HM574660 HM574443; *I. kopetdagensis* (Vved.) B.Mathew & Wendelbo FR870433 FR870433 FR870644 FR870644 FR870710 FR870578; *I. koreana* Nakai FJ156308 FJ197285 FJ197238 EU939479 EU939479 HM574444; *I. kuschakewiczii* B.Fedtsch. HM574518 FR870430 FR870430 FR870641 FR870641 FR870707 FR870575; *I. lactea* Pall. FJ156309 FJ197286 FJ197239 EU939480 EU939480 HM574446; *I. lacustris* Nutt. HM574567 HM574692 HM574447; *I. laevigata* Fisch. FJ156310 FJ197287 FJ197240 EU939481 EU939481; *I. lazica* Albov AY596626 AY596626 EU939482 EU939482 HM574448; *I. leptorrhiza* Vved. FR870408 FR870408 FR870619 FR870619 FR870685 FR870553; *I. linifolia* O.Fedtsch. FR870378 FR870378 FR870589 FR870589 FR870655 FR870523; *I. loczyi* Kanitz JF954158 AF480397 AF480375; *I. longipetala* Herb. FJ156313 FJ197289 FJ197242 EU939484 EU939484 HM574449; *I. macrosiphon* Torr. FJ156314 FJ197290 FJ197243 EU939485 EU939485; *I. magnifica* Vved. FR870406 FR870406 FR870617 FR870617 HM574450 FR870683 FR870551; *I. mandshurica* Maxim. HM574516 HM574643 HM574451; *I. maracandica* (Vved.) Wendelbo FR870420 FR870420 FR870631 FR870631 FR870697 FR870565; *I. masia* Dykes FJ156315 AY596628 AY596628 EU939486 EU939486 HM574452; *I. microglossa* Wendelbo FR870404 FR870404 FR870615 FR870615 FR870681 FR870549; *I. milesii* Baker ex Foster FR870424 FR870424 FR870635 FR870635 FR870701 FR870569; *I. minutoaurea* Makino FJ156316 FJ197291 FJ197244 EU939487 EU939487 HM574453; *I. missouriensis* Nutt. FJ156317 AY596648 AY596648 EU939488 EU939488 AF547003; *I. munzii* R.C.Foster FJ156318 FJ197292 FJ197245 EU939489 EU939489 HM574455; *I. musulmanica* Fomin AY596634 AY596634; *I. narbutii* O.Fedtsch. FR870388 FR870388 FR870599 FR870599 FR870665 FR870533; *I. narynensis* O.Fedtsch. FR870380 FR870380 FR870591 FR870591 FR870657 FR870525; *I. nectarifera* Güner HM574507 HM574634 HM574456; *I. nezahatiae* Güner & H.Duman FR870382 FR870382 FR870593 FR870593 FR870659 FR870527; *I. nicolai* Vved. FR870379 FR870379 FR870590 FR870590 FR870656 FR870524; *I. nigricans* Dinsm. HM574551 HM574457; *I. notha* M.Bieb. AY596633 AY596634; *I. nusairiensis* Mouterde FR870376 FR870376 FR870587 FR870587 FR870653 FR870521; *I.* aff. *nusairiensis* Mouterde FR870422 FR870422 FR870633 FR870633 FR870699 FR870567; *I. odaesanensis* Y.N.Lee FJ156319 FJ197293 FJ197246 EU939490 EU939490 HM574458; *I. orientalis* Mill. FJ156320 AY596636 AY596636 EU939491 EU939491 HM574460; *I. palaestina* Boiss. FR870387 FR870387 FR870598 FR870598 FR870664 FR870532; *I. pallasii* Fisch. ex Trevir. AF480399 AF480377; *I. pallida* Lam. HM574564 HM574689 HM574461; *I. pamphylica* Hedge HM574568 HM574693 HM574462; *I. parvula* (Vved.) Vved. FR870399 FR870399 FR870610 FR870610 FR870676 FR870544; *I.* aff. *parvula* (Vved.) Vved. FR870427 FR870427 FR870638 FR870638 FR870704 FR870572; *I. persica* L. HM574530 FR870383 FR870383 FR870594 FR870594 FR870660 FR870528; *I. planifolia* (Mill.) Fiori & Paol. HM574531 HM574658 HM574597 FR870621 FR870621 HM574464 FR870687 FR870555 ; *I. popovii* Vved. FR870415 FR870415 FR870626 FR870626 FR870692 FR870560; *I. postii* Mouterde FR870375 FR870375 FR870586 FR870586 FR870652 FR870520; *I. potaninii* Maxim. HM574515 AY596654 AY596654 HM574466; *I. potaninii* Maxim. var. *ionanthus* Y.T.Zhao HM574514 HM574641 HM574465; *I. prismatica* Pursh FJ156321 FJ197294 FJ197247 EU939492 EU939492 HM574467; *I. proantha* Diels var. *valida* (S.S.Chien) Y.T.Zhao HM574565 HM574690 HM574468; *I. pseudacorus* L. FJ156322 AY596643 AY596643 EU939493 EU939493 HM574469; *I. pseudocapnoides* Ruksans FR870391 FR870391 FR870602 FR870602 FR870668 FR870536; *I. pseudocaucasica* Grossh. FR870394 FR870394 FR870605 FR870605 FR870671 FR870539; *I. pumila* L. HM574510 HM574470; *I. purdyi* Eastw. FJ156323 FJ197295 FJ197248 EU939494 EU939494 HM574471; *I. purpureobractea* B.Mathew & T.Baytop HM574511 HM574638 HM574472; *I. regis-uzziae* Feinbrun FR870409 FR870409 FR870620 FR870620 FR870686 FR870554; *I. reticulata* M.Bieb. AY596629 HM574600 HM574473; *I. rosenbachiana* Regel FR870411 FR870411 FR870622 FR870622 FR870688 FR870556; *I. ruthenica* Ker Gawl. FJ156324 FJ197296 FJ197249 EU939495 EU939495; *I. sanguinea* Hornem. ex Donn. FJ156325 FJ197297 FJ197250 EU939496 EU939496 HM574474; *I. sari* Schott ex Baker FJ156326 AY596659 AY596659 EU939497 EU939497 HM574475; *I. schachtii* Markgr. AY596663 AY596663; *I. setosa* Pall. ex Link 1 FJ156327 AY596644 AY596644 EU939498 EU939498 HM574476; *I. setosa* Pall. ex Link 2 KC118939; *I. sibirica* L. FJ156328 AY596642 AY596642 EU939499 EU939499 HM574477; *I. sogdiana* Bunge FJ156329 FJ197298 FJ197251 EU939500 EU939500; *I. songarica* Schrenk FJ156330 FJ197299 FJ197252 EU939501 EU939501 HM574478; *I. sprengeri* Siehe FJ156331 FJ197300 FJ197253 EU939502 EU939502 HM574479; *I. spuria* L. HM574545 AY596635 HM574610 HM574480; *I. staintonii* Hara FR870423 FR870423 FR870634 FR870634 FR870700 FR870568; *I. stenophylla* Hausskn. ex Baker FR870398 FR870398 FR870609 FR870609 FR870675 FR870543; *I. stenophylla* Hausskn. subsp. *allisonii* B.Mathew FR870401 FR870401 FR870612 FR870612 FR870678 FR870546; *I. stocksii* Hemsl. & Lace FR870374 FR870374 FR870585 FR870585 FR870651 FR870519; *I. subbiflora* Brot. FJ156333 FJ197301 FJ197254 EU939504 EU939504 HM574482 FR870651 FR870519; *I.*

subdecolorata **Vved. 1 FR870414 FR870414 FR870623 FR870623 FR870691 FR870557; *I. subdecolorata* Vved. 2 FR870412 FR870625 FR870625 FR870559; *I. svetlanae* (Vved.) T.Hall & Seisums FR870395 FR870395 FR870606 FR870606 FR870672 FR870540; *I. swensoniana* Chaudhary, G.Kirkw. & C.Weymouth HM574506 HM574633 HM574483; *I. tadshikorum* Vved. FR870403 FR870403 FR870614 FR870614 FR870680 FR870548; *I. taochia* Woronow ex Grossh. FJ156334 FJ197302 FJ197255 EU939505 EU939505 HM574484; *I. tectorum* Maxim. FR870435 FR870435 FR870646 FR870646 FR870712 FR870580; *I. tenax* Douglas ex Lindl. *klamathensis* L.W.Lenz FJ197303 FJ197256 EU939506 EU939506; *I. tenuifolia* Pall. JF954184 AF480406 AF480384; *I. tenuis* S.Watson FJ156337 AY596638 AY596638 EU939508 EU939508 HM574486; *I. tenuissima* Dykes FJ156338 FJ197305 FJ197257 EU939510 EU939510 HM574487; *I. tenuissima* Dykes subsp. *purdyiformis* (R.C.Foster) L.W.Lenz FJ156339 FJ197304 FJ197258 EU939509 EU939509; *I. thompsonii* (R.C.Foster) L.W.Lenz FJ156340 FJ197306 FJ197259 EU939511 EU939511; *I. tigridia* Bunge AY596653 HM574613 HM574488; *I. tingitana* Boiss. & Reut. FJ156341 AY596627 AY596627 EU939512 EU939512 HM574489; *I. tridentata* Pursh FJ156342 FJ197260 EU939513 EU939513 HM574490; *I. tubergeniana* Foster FR870396 FR870396 FR870607 FR870607 FR870673 FR870541; *I.* aff. *tubergeniana* Foster FR870437 FR870437 FR870648 FR870648 FR870714 FR870582; *I. tuberosa* L. HM574539 HM574666 HM574491; *I. typhifolia* Kitag. FJ156343 FJ197308 FJ197261 EU939514 EU939514 HM574492; *I. unguicularis* Poir. FJ156344 AY596625 AY596625 EU939515 EU939515 HM574493; *I. uniflora* Pall. ex Link FJ156345 FJ197309 FJ197262 EU939516 EU939516 HM574494; *I. ventricosa* Pall. AF480409 AF480387; *I. verna* L. FJ156346 FJ197310 FJ197263 EU939517 EU939517; *I. versicolor* Thunb. FJ156347 KC118948 FJ197264 EU939518 EU939518; *I. vicaria* (Vved.) Vved. FR870393 FR870393 FR870604 FR870604 FR870670 FR870538; *I.* aff. *vicaria* (Vved.) Vved. FR870438 FR870438 FR870649 FR870649 FR870715 FR870583; *I. virginica* L. FJ156349 AY596641 FJ197266 EU939520 EU939520 HQ181116; *I. virginica* L. var. *shrevei* (Small) E.S.Anderson FJ156348 AY596641 FJ197265 EU939519 EU939519 HM574495; *I. warleyensis* Foster FR870397 FR870397 FR870608 FR870608 FR870674 FR870542; *I. wattii* Baker ex Hook.f. HM574536 AY596640 HM574602 HM574496; *I. willmottiana* Foster FR870407 FR870407 FR870618 FR870618 FR870684 FR870552; *I. wilsonii* C.H.Wright FJ156350 FJ197314 FJ197267 EU939521 EU939521 HM574497; *I. winogradowii* Fomin HM574535 HM574662 HM574498; *I. xiphium* L. HM574524 HM574651 HM574499; *I. zaprjagajevii* (N.V.Abramov) T.Hall & Seisums FR870405 FR870405 FR870616 FR870616 FR870682 FR870550**

Outgropus: ***Dietes robinsoniana*** **Klatt HM574394; *Gladiolus caucasicus* Herb. AY596622 AY596622 EU939454 EU939454 HM574395; *Gynandriris pritzeliana* (Diels) Goldblatt FJ156351 AY596623 EU939522 EU939522 HM574500; *G. sisyrinchium* Parl. AY596624 AY596624 AJ307299; *Tigridia pavonia* (L.f.) DC. AY225087; *Trillium ovatum* Pursh DQ826350 AY727466**

Sources of sequence data: AB088786: Tamura et al., 2010; **AF********: Makarevitch et al., 2003; **AF547003, AY225087**: Givnish et al., 2005; **AJ307299**: Goldblatt et al., 2002; **AJ********: Goldblatt et al., 2008; **AY5*******: Wilson, 2004; **AY727466:** Shaw et al, 2005; **DQ826350**: Shaw et al, 2007; **EU9******* & **FJ1*******: Wilson, 2009 **FR********: Ikinci et al., 2012; **HE967421**: Bruni et al., 2012; **HM********: Wilson, 2011; **HQ181116**: Givnish et al., 2012; **JF********: Li et al., 2011; **JN895312**: de Vere et al., 2012; **K*********: Wheeler & Wilson, 2013, unpubl.

***In many cases, from one to a few neighboring loci be submitted under the same accession number, therefore the same accession number may represent one or a few loci and listed in the Table for more than one time (like, for example number FR870613 (*Iris aitchisonii*) listed twice for both *trnL* gene and trnL-F IGS).**
